# Risk factors and medical resource utilization in US adults hospitalized with influenza or respiratory syncytial virus in the Hospitalized Acute Respiratory Tract Infection study

**DOI:** 10.1111/irv.12994

**Published:** 2022-04-26

**Authors:** Jessica Hartnett, Prina Donga, Gabriela Ispas, Yannick Vandendijck, David Anderson, Stacey House, Selim Suner

**Affiliations:** ^1^ Janssen Scientific Affairs, LLC Titusville New Jersey USA; ^2^ Janssen Research & Development Beerse Belgium; ^3^ Department of Emergency Medicine Washington University School of Medicine St. Louis Missouri USA; ^4^ Department of Emergency Medicine Alpert Medical School of Brown University Providence Rhode Island USA

**Keywords:** hospitalization, influenza, prospective study, respiratory infections, respiratory syncytial virus, risk factors

## Abstract

**Background:**

Influenza and respiratory syncytial virus (RSV) are associated with substantial morbidity and mortality in the United States. We assessed risk factors for severe disease and medical resource utilization (MRU) among US adults hospitalized with influenza or RSV in the Hospitalized Acute Respiratory Tract Infection (HARTI) study.

**Methods:**

HARTI was a prospective global (40 centers, 12 countries) epidemiological study of adults hospitalized with acute respiratory tract infections conducted across the 2017–2019 epidemic seasons. Patients with confirmed influenza or RSV were followed up to 3 months post‐discharge. Baseline characteristics, prevalence of core risk factors (CRFs) for severe disease (age ≥65 years, chronic heart or renal disease, chronic obstructive pulmonary disease, or asthma), and MRU were summarized descriptively.

**Results:**

The US cohort included 280 influenza‐positive and 120 RSV‐positive patients. RSV patients were older (mean: 63.1 vs. 59.7 years) and a higher proportion had CRFs (87.5% vs. 81.4%). Among those with CRFs (influenza, n = 153; RSV, n = 99), RSV patients required longer hospitalizations (median length of stay: 4.5 days) and a greater proportion (79.8%) required oxygen supplementation during hospitalization compared with influenza patients (4.0 days and 59.5%, respectively). At 3 months post‐discharge, a greater proportion of RSV patients with CRFs reported use of antibiotics, antitussives, bronchodilators, and inhaled and systemic steroids versus those with influenza and CRFs. Many patients with CRFs reported hospital readmission at 3 months post‐discharge (RSV: 13.4%; influenza: 11.9%).

**Conclusions:**

MRU during and post‐hospitalization due to RSV in adults is similar to or greater than that of influenza. Enhanced RSV surveillance and preventive and therapeutic interventions are needed.

## 1 BACKGROUND

Lower respiratory tract infections (LRTIs) cause substantial morbidity and mortality globally. In 2016, an estimated 2.38 million LRTI‐associated deaths occurred across all age groups worldwide.^1^ Influenza and respiratory syncytial virus (RSV) are among the most common causes of LRTIs during typical (i.e., non‐pandemic) endemic seasons,^2^ with influenza causing an estimated 1 billion infections^3^ and RSV causing an estimated 64 million acute respiratory infections^4^ globally each year. A systematic analysis from the Global Burden of Diseases, Risk Factors, and Injuries study found that influenza caused ~500,000 deaths and RSV caused 250,000 deaths annually worldwide.^5^ In the United States, there are an estimated 50,000 influenza‐associated and 17,000 RSV‐associated deaths each year.^6^ The annual disease burden of both influenza and RSV within the United States varies substantially by geographic region^7^, ^8^ and endemic season.^9^, ^10^ Importantly, RSV‐associated burden may be underestimated due to a lack of routine testing for RSV and low provider awareness.^11^

Children (<1 year) and older adults (≥65 years) comprise the majority of RSV‐associated hospitalizations.^12^ A retrospective study analyzing New York City hospitalization data in infants aged <1 year and children aged 1–4 years reported annual average influenza‐associated hospitalization rates of 129.0 and 36.4 per 100,000 population and RSV‐associated hospitalization rates of 1895.8 and 116.7 per 100,000 population, respectively.^13^ Influenza and RSV also cause substantial mortality in infants aged <1 year (2.2 and 5.4 per 100,000 person‐years, respectively) and children aged 1–4 years (1.1 and 0.9 per 100,000 person‐years, respectively).^6^ Older and high‐risk adults (i.e., immunocompromised individuals and those with underlying respiratory or cardiovascular comorbidities^14^, ^15^) also experience high RSV‐associated morbidity and mortality. Hospitalization rates for high‐risk adults of all ages infected with influenza (242 per 100,000 population) and RSV (91 per 100,000 population) are comparable with those for older adults of all risk levels (256 and 84 per 100,000 population among adults aged 65–74 years, respectively).^16^ A prospective study of hospitalized adults in Nashville, TN, found that both influenza (11.8 per 10,000 residents) and RSV (15.0 per 10,000 residents) cause substantial hospitalization rates among adults aged ≥50 years^17^; the majority of these hospitalizations are concentrated during seasonal surges. The estimated mortality rate among US adults aged ≥65 years is 132.5 and 29.6 per 100,000 person‐years for influenza and RSV, respectively.^6^

LRTIs also require significant medical resource utilization (MRU). In the United States, influenza is responsible for an estimated 3.1 million hospitalized days, 31.4 million outpatient visits, and $87.1 billion in total economic burden annually.^18^ A recent analysis using data from published studies and public health databases estimated the average cost of influenza‐associated hospitalization in the United States ranged from $5211 (in patients <1 year of age) to $12,102 (in patients aged 45–64 years), depending on age group.^19^ There are limited data on RSV‐specific MRU. In a recent retrospective claims study among hospitalized adults in New York state, the average cost of an RSV‐associated hospitalization was $8403, and the total annual cost of RSV‐associated hospitalizations in the United States was estimated at $1.2 billion.^20^ Additionally, in a different community cohort study in New York City, among RSV‐positive hospitalized adults aged ≥65 years, the majority received chest X‐rays, antibiotics, and steroids; the mean length of hospital stay in this population was 6.6 days.^12^ Seasonal LRTI outbreaks cause significant emergency department utilization, crowding, and potential hospital staff shortages due to infections among staff or their families.^21^

Older adults, immunocompromised individuals, and those with underlying respiratory or cardiovascular comorbidities are at risk for severe influenza‐ or RSV‐mediated disease.^14^, ^15^ Detailed assessments of the characteristics of adults hospitalized with influenza or RSV and MRU during and post‐hospitalization are lacking^17^; such assessments are important for improving diagnostics, surveillance, prophylaxis, and treatments for both pathogens.

The Hospitalized Acute Respiratory Tract Infection (HARTI) study was a global study during the 2017–2019 epidemic seasons aimed at assessing risk factors for severe disease and MRU in adults hospitalized with acute respiratory tract infections (ARTIs).^15^ We used data from the US cohort of HARTI to assess risk factors for severe disease and MRU in adults hospitalized with influenza or RSV.

## 2 METHODS

### 2.1 Study design

**FIGURE 1:**
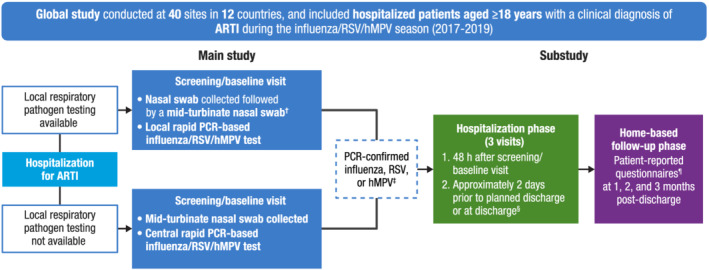
Study design. ADL, activities of daily living; ARTI, acute respiratory tract infection; EQ‐5D‐5L, EuroQol 5 Dimensions 5 Levels; hMPV, human metapneumovirus; IADL, instrumental activities of daily living; MRU, medical resource utilization; PCR, polymerase chain reaction; RiiQ™, Respiratory Intensity and Impact Questionnaire; RSB, Respiratory Symptoms Bother and Change in Health Status Questionnaire; RSV, respiratory syncytial virus; SoC, standard‐of‐care. ^†^When a nasal swab was collected as part of SoC, a mid‐turbinate swab was collected from the opposite nostril and then used for the SoC test. Rapid PCR analysis was used to identify respiratory pathogens from the SoC nasal and mid‐turbinate swabs. ^‡^Leftover nasal swab or blood samples were stored for potential future exploratory research. ^§^For patients discharged within 48 h of screening, only one visit was conducted (at discharge). ^¶^Patient‐reported questionnaires included the Barthel ADL, Lawton IADL, RiiQ™, RSB, EQ‐5D‐5L, and MRU questionnaires.

HARTI was a prospective cohort study in adults (≥18 years old) hospitalized with ARTIs during the influenza/RSV/human metapneumovirus (hMPV) season at 40 centers across 12 countries (Australia, Argentina, Brazil, Canada, France, Germany, Japan, Malaysia, Mexico, Republic of Korea, South Africa, and the United States) over two consecutive epidemic seasons (2017–2019; Figure 1). The US portion of the study was conducted in five centers (Rochester, NY; Augusta, GA; Iowa City, IA; Providence, RI; and St. Louis, MO). The enrollment period was based on local respiratory virus epidemic wave progression; US cohort patients were enrolled from March 07, 2017, to April 16, 2019. Patients provided consent and were enrolled in the study within 24 h after hospital admission. Clinical ARTI diagnoses and hospitalization decisions were made according to local standard‐of‐care (SoC) practices. Viral testing (reverse transcription polymerase chain reaction [RT‐PCR]) was conducted either as part of SoC practices or by study‐specific molecular diagnostic testing with approved diagnostic platforms if not performed as part of SoC. Patients with confirmed influenza, RSV, or hMPV (latter not included in this analysis due to small sample size) were invited to enroll in the substudy comprising a hospitalization phase (two visits: 48 h post‐enrollment, and within 2 days prior to discharge) and a follow‐up phase with phone interviews at 1, 2, and 3 months post‐discharge.

### 2.2 Data collection

Clinical information and MRU were collected through clinician‐reported questionnaires (at enrollment, 48 h post‐enrollment, and within 2 days prior to discharge) and through MRU questionnaires during monthly patient phone interviews post‐discharge. If a patient was discharged within 3 days of enrollment, only one hospital assessment was performed after enrollment (at discharge). Vital signs and complications (lower respiratory complications, cardiovascular complications, bacterial superinfections, and other complications) were documented during hospitalization. The MRU questionnaire assessing hospital readmissions (emergency department, general ward, or critical care), the use of medical consultations (pulmonologist, respiratory physiotherapist, or other), professional home care (general practitioner, nurse, respiratory physiotherapist, or other), and medications taken (antibiotics, bronchodilators, inhaled steroids, systemic steroids, antitussives, oxygen, or other) was administered at 1, 2, and 3 months post‐discharge.

### 2.3 Statistical analysis

Data collected from study questionnaires were summarized descriptively by pathogen and risk group. Patients were considered to have core risk factors (CRFs) for progression to severe disease if they were aged ≥65 years or had chronic heart disease, chronic renal disease, chronic obstructive pulmonary disease (COPD), or asthma.

Clinical symptom severity was assessed at screening (within 24 h of hospitalization) using the National Early Warning Score (NEWS), a validated tool used to detect clinical deterioration in adults, which is calculated using seven graded vital sign measurements (respiratory rate, oxygen saturation, oxygen supplementation, temperature, blood pressure, heart rate, and level of consciousness).^22^, ^23^, ^24^, ^25^ Each vital sign was scored from 0 to 3; NEWS scores were calculated by summing vital sign scores, with higher scores representing more severe illness (low severity: 0–4; moderate: 5–6 or an individual parameter scoring of 3; high: ≥7). Since all patients signed the informed consent form for study participation, level of consciousness was assumed to be “Alert” (i.e., a score of 0) for all patients at enrollment.

Symptom severity was also assessed using a total clinical symptom score comprising general symptom (cough, sputum production, shortness of breath, and malaise), lower respiratory symptom (dyspnea; rales, rhonchi, or other abnormal breath sounds; and wheezing), and upper respiratory symptom (nasal discharge, pharyngitis, and sinus tenderness) scores, with higher scores representing more severe disease (see Table S1). Hospital length of stay (LoS) was summarized and reported categorically by ≤3 or >3 days.

## 3 RESULTS

### 3.1 Patients

**FIGURE 2:**
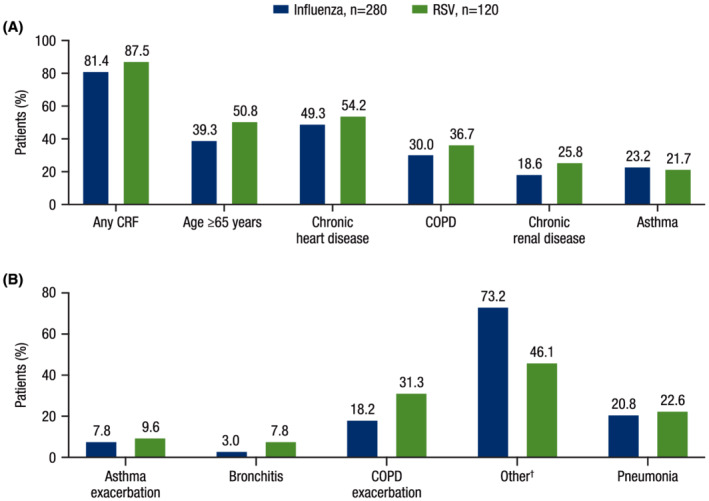
Baseline CRFs and ARTIs leading to hospitalization (main study). (A) Percentage of patients with CRFs at baseline and (B) ARTIs reported by patients leading to hospitalization. ARTI, acute respiratory tract infection; COPD, chronic obstructive pulmonary disease; CRF, core risk factor; RSV, respiratory syncytial virus. ^†^Other ARTIs primarily consisted of viral influenza‐like ARTIs.

The US cohort of the international HARTI study included 280 patients with influenza and 120 patients with RSV in the main study (Table 1). Patients with RSV were older (mean [standard deviation (SD)] age: 63.1 [15.9] years) than patients with influenza (59.7 [16.9] years). Patients with RSV reported greater symptom severity at hospitalization as measured by NEWS (mean [SD]: 4.3 [2.6]) and total clinical symptom scores (16.1 [5.6]) compared with patients with influenza (3.4 [2.5] and 14.9 [5.4], respectively). The majority of patients had CRFs (RSV: 87.5%; influenza: 81.4%; Figure 2A). A greater percentage of patients with RSV had additional concurrent medical conditions during hospitalization, including acute exacerbation of asthma or COPD, exacerbation of congestive heart failure (CHF), and hypoxemia (33.3%, 22.7%, and 34.7%, respectively) compared with patients with influenza (24.4%, 16.5%, and 25.0%, respectively; Figure 2B). Oxygen supplementation prior to hospitalization was only collected for patients with COPD; among these, a higher proportion of those with RSV had at‐home supplemental oxygen use at baseline compared with those with influenza (Table S2).

**TABLE 1:** Participant demographic and baseline characteristics (main study)

	Influenza (n = 280)	RSV (n = 120)
Age, mean (SD), years	59.7 (16.9)	63.1 (15.9)
18 to ≤59 years, n (%)	130 (46.4)	47 (39.2)
60 to ≤64 years, n (%)	40 (14.3)	12 (10.0)
65 to ≤74 years, n (%)	60 (21.4)	34 (28.3)
≥75 years, n (%)	50 (17.9)	27 (22.5)
Female, n (%)	149 (53.2)	81 (67.5)
Acute respiratory symptom length before hospitalization, mean (SD), days	4.2 (4.1)	5.5 (4.8)
≤3 days, n (%)	160 (57.1)	50 (41.7)
>3 days, n (%)	120 (42.9)	70 (58.3)
Additional concurrent medical conditions during hospitalization,^a^ n (%)	n = 164	n = 75
Acute exacerbation of asthma or COPD	40 (24.4)	25 (33.3)
Any transplantation	1 (0.6)	1 (1.3)
Exacerbation of congestive heart failure	27 (16.5)	17 (22.7)
Hypoxemia	41 (25.0)	26 (34.7)
Malignancy or need for diagnostic workup	4 (2.4)	4 (5.3)
Sepsis	35 (21.3)	11 (14.7)
O_2_ supplementation at screening visit, n (%)	133 (47.5)	86 (71.7)
NEWS scale at screening,^b^ mean (SD)	3.4 (2.5)	4.3 (2.6)
Total clinical symptom score, mean (SD)^c^	14.9 (5.4)	16.1 (5.6)
18 to ≤59 years^d^	14.8 (5.4)	15.1 (6.1)
60 to ≤64 years^e^	16.2 (4.5)	19.2 (4.5)
65 to ≤74 years^f^	14.3 (5.8)	16.7 (5.5)
≥75 years^g^	14.5 (5.4)	15.5 (5.1)
Residence pre‐hospitalization, n (%)		
LTCF	2 (0.7)	4 (3.3)
Private residence	256 (91.4)	110 (91.7)
Retirement community	5 (1.8)	2 (1.7)
Other	11 (3.9)	2 (1.7)
Not reported	6 (2.1)	2 (1.7)

Abbreviations: COPD, chronic obstructive pulmonary disease; LTCF, long‐term care facility; NEWS, National Early Warning Score; RSV, respiratory syncytial virus; SD, standard deviation.

^a^ Additional concurrent medical conditions during hospitalization are not inclusive of one another. Responses with multiple conditions were allowed.

^b^ Data available for 271/280 patients with influenza and 109/120 patients with RSV.

^c^ Data available for 273/278 patients with influenza and 119/120 patients with RSV.

^d^ Data available for 130/130 patients with influenza and 47/47 patients with RSV.

^e^ Data available for 39/40 patients with influenza and 12/12 patients with RSV.

^f^ Data available for 57/60 patients with influenza and 34/34 patients with RSV.

^g^ Data available for 47/50 patients with influenza and 26/27 patients with RSV.

### 3.2 MRU

MRU during and post‐hospitalization were assessed in the substudy, which enrolled 178 and 112 patients with influenza and RSV, respectively; of these, 153 patients (86.0%) with influenza and 99 patients (88.4%) with RSV had CRFs.

**FIGURE 3:**
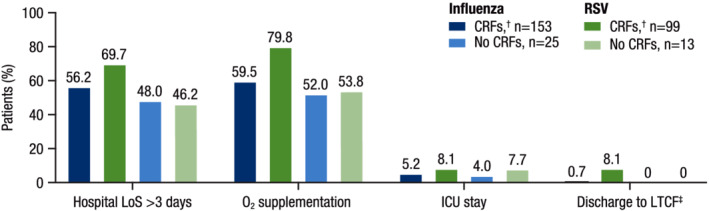
Key MRU parameters during hospitalization. CRF, core risk factor; COPD, chronic obstructive pulmonary disease; ICU, intensive care unit; LoS, length of stay; LTCF, long‐term care facility; RSV, respiratory syncytial virus. ^†^CRFs included any one or a combination of the following: age ≥65 years, chronic heart disease, COPD, chronic renal disease, and asthma. ^‡^The one patient with influenza discharged to a LTCF was in an LTCF prior to hospitalization. Of the eight patients with RSV who were discharged to LTCFs, four patients reported LTCF residence prior to hospitalization.

During hospitalization, patients with RSV required greater MRU compared with patients with influenza (Table 2). Regardless of the presence of CRFs, a greater percentage of patients with RSV required intensive care unit (ICU) stays during hospitalization (without CRFs: 7.7%; with CRFs: 8.1%) compared with patients with influenza (without CRFs: 5.2%; with CRFs: 4.0%). Among patients with CRFs, those with RSV required longer hospital stays (median [range] hospital LoS: 4.5 [2, 17] days) and a greater proportion (79.8%) required supplemental oxygen compared with those with influenza (4 [1, 18] days and 59.5%, respectively). Among patients with RSV, a greater proportion of those with CRFs (79.8%) required oxygen supplementation during hospitalization compared with those without CRFs (53.8%); among patients with influenza, similar proportions of patients required oxygen supplementation (with CRFs: 59.5%; without CRFs: 52.0%). A greater proportion of patients with RSV and CRFs (69.7%) required hospital stays >3 days compared with patients with influenza and CRFs (56.2%; Figure 3). Patients with RSV also experienced more complications during hospitalization, including lower respiratory complications, cardiovascular complications, and bacterial superinfections, compared with patients with influenza (Figure S1).

**TABLE 2:** MRU during hospitalization (substudy)

	CRFs^a^		No CRFs	
	Influenza (n = 153)	RSV (n = 99)	Influenza (n = 25)	RSV (n = 13)
Length of hospital stay,^b^ mean (SD), days	4.7 (3.0)^c^	5.4 (3.1)^d^	4.8 (4.6)	6.7 (10.1)
Median (range), days	4 (1, 18)	4.5 (2, 17)	3 (2, 21)	3 (1, 38)
≤3 days, n (%)	67 (43.8)	30 (30.3)	13 (52.0)	7 (53.8)
>3 days, n (%)	86 (56.2)	69 (69.7)	12 (48.0)	6 (46.2)
O_2_ supplementation given, n (%)	91 (59.5)	79 (79.8)	13 (52.0)	7 (53.8)
O_2_ supplementation duration, mean (SD), days	4.5 (3.6)^e^	4.2 (2.8)^f^	4.1 (4.3)^g^	4.2 (4.6)^h^
Mechanical ventilation given, n (%)	3 (2.0)	0 (0)	0 (0)	0 (0)
ICU stay during hospitalization, n (%)	8 (5.2)	8 (8.1)	1 (4.0)	1 (7.7)
ICU stay duration, mean (SD), days	4.8 (1.3)	2.5 (1.0)^i^	3.0 (−)	4.0 (−)
ICU stay duration, median (range), days	4.5 (3, 7)	2 (2, 4)^i^	3 (3, 3)	4 (4, 4)

Abbreviations: COPD, chronic obstructive pulmonary disease; CRF, core risk factor; ICU, intensive care unit; MRU, medical resource utilization; RSV, respiratory syncytial virus; SD, standard deviation.

^a^ CRFs included any one or a combination of the following: age ≥65 years, chronic heart disease, COPD, chronic renal disease, and asthma.

^b^ Data from four subjects with censored length of hospital stay data not included in mean (SD) and median (range) results. Censored data included three patients with influenza and CRFs (>2, >3, and >6 days) and one patient with RSV and CRFs (<3 days).

^c^ Data available for 150/153 patients.

^d^ Data available for 98/99 patients.

^e^ Data available for 82/91 patients given supplemental oxygen.

^f^ Data available for 73/79 patients given supplemental oxygen.

^g^ Data available for 13/13 patients given supplemental oxygen.

^h^ Data available for 6/7 patients given supplemental oxygen.

^i^ Data available for 4/8 patients with ICU stays during hospitalization.

**FIGURE 4:**
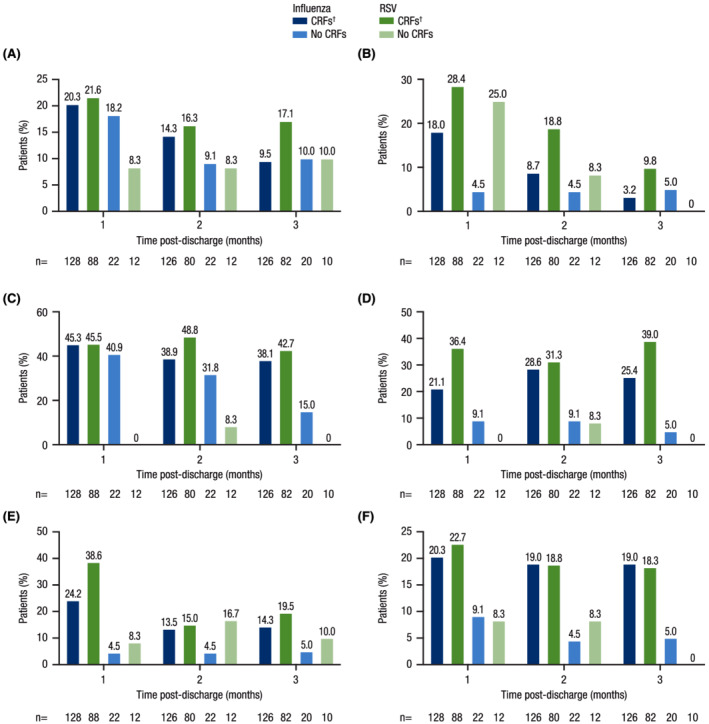
Medication use post‐discharge (substudy). Percentage of patients reporting use of (A) antibiotics, (B) antitussives, (C) bronchodilators, (D) inhaled steroids, (E) systemic steroids, or (F) oxygen at 1, 2, and 3 months post‐discharge. CRF, core risk factor; COPD, chronic obstructive pulmonary disease; RSV, respiratory syncytial virus. ^†^CRFs included any one or a combination of the following: age ≥65 years, chronic heart disease, COPD, chronic renal disease, and asthma.

**FIGURE 5:**
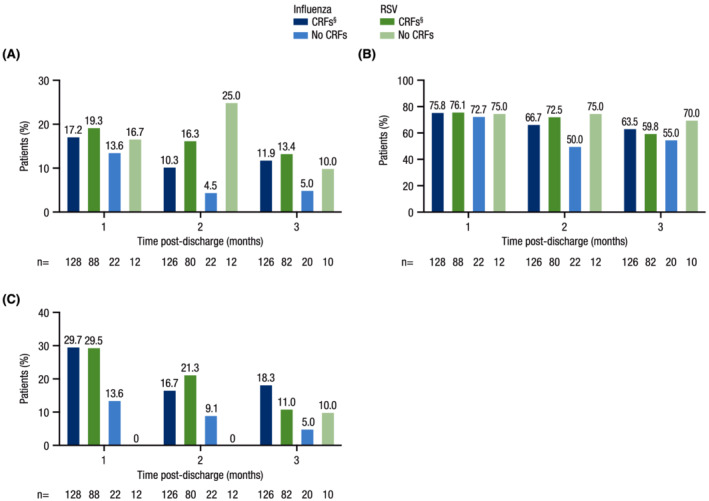
MRU post‐discharge (substudy). Percentage of patients reporting (A) hospital readmission, (B) use of medical consultations,^†^ and (C) professional home care^‡^ at 1, 2, and 3 months post‐discharge. CRF, core risk factor; COPD, chronic obstructive pulmonary disease; MRU, medical resource utilization; RSV, respiratory syncytial virus. ^†^Medical consultations included utilization of a pulmonologist or respiratory physiotherapist. ^‡^Professional home care included utilization of a general practitioner, nurse, or respiratory physiotherapist. ^§^CRFs included any one or a combination of the following: age ≥65 years, chronic heart disease, COPD, chronic renal disease, and asthma.

In general, patients with CRFs (regardless of pathogen) reported more medication use at 1, 2, and 3 months post‐discharge compared with patients without CRFs; those with RSV and CRFs generally reported more medication use post‐discharge than those with influenza and CRFs (Figure 4). Medication use and MRU at 3 months post‐discharge were available for 126 patients with influenza and CRFs and 82 patients with RSV and CRFs. At 3 months post‐discharge, a greater proportion of patients with RSV and CRFs compared with patients with influenza and CRFs reported use of concomitant medications including antibiotics (17.1% vs. 9.5%, respectively), antitussives (9.8% vs. 3.2%), bronchodilators (42.7% vs. 38.1%), and inhaled (39.0% vs. 25.4%) and systemic (19.5% vs. 14.3%) steroids. Oxygen use at 3 months post‐discharge was reported by similar proportions of patients with CRFs, regardless of pathogen (influenza: 19.0%; RSV: 18.3%). Patients with CRFs reported substantial hospital readmission rates (influenza: 11.9%; RSV: 13.4%), use of medical consultations (influenza: 63.5%; RSV: 59.8%), and use of professional home care (influenza: 18.3%; RSV: 11.0%) at 3 months post‐discharge (Figure 5).

## 4 DISCUSSION

In the US cohort of HARTI, RSV was associated with MRU comparable with or greater than influenza. Observations in the US cohort reflect those in the global HARTI population; patients with RSV were older and a greater proportion had CRFs compared with patients with influenza.^15^ An analysis using data from the global HARTI study also found that patients with RSV reported more severe lower respiratory tract symptoms up to 3 months post‐discharge compared with patients with influenza, in agreement with results in this study showing that patients with RSV reported greater MRU at 3 months post‐discharge compared with those with influenza.^26^ Additionally, results from the US cohort of HARTI are also consistent with other studies comparing the disease burden of RSV and influenza. In a retrospective claims study among hospitalized adults aged ≥60 years, Ackerson *et al* found that patients with RSV were older, more likely to have CHF and COPD at baseline, and had greater odds of requiring a hospital stay ≥7 days and ICU admission.^27^ Another retrospective claims study found that, among hospitalized adults in New York City, patients with RSV required a longer hospital LoS than patients with influenza (median: 4.4 vs. 3.9 days, respectively).^28^ Patients with RSV also more frequently experienced acute exacerbation of asthma or COPD, exacerbation of CHF, and hypoxemia; this may parallel the association between RSV infections early in life and development of bronchospastic diseases such as asthma and recurrent wheezing in childhood.^29^, ^30^

Collectively, these results suggest that the severity of RSV‐associated illness in adults rivals that of influenza. However, there is currently no licensed RSV vaccine, and there are only two US Food and Drug Administration–approved antiviral drugs for either RSV prophylaxis (palivizumab) or treatment (ribavirin).^31^ Palivizumab, though effective at preventing RSV infections, is only indicated for pediatric populations (specifically those aged ≤24 months^32^), and ribavirin has limited efficacy due to its non‐specific antiviral activity, constraining providers' ability to alleviate RSV‐associated disease burden.^31^ Two other monoclonal antibody treatments are also currently in development; nirsevimab^33^, ^34^ is currently under development for use in term, pre‐term, and high‐risk infants, and clesrovimab^35^ is also being investigated for use in infants and neonates. In contrast, there are vaccines against influenza and four different antiviral drugs (peramivir, zanamivir, oseltamivir phosphate, and baloxavir marboxil) approved for use in adults in the United States.^36^, ^37^

In this study, differences between adults hospitalized with influenza and those hospitalized with RSV were particularly evident among patients with CRFs. Among patients with CRFs, a greater proportion of those with RSV had hospital LoS >3 days, required oxygen supplementation, and had ICU stays during hospitalization compared with those with influenza (Figure 3). Additionally, among patients with CRFs, those with RSV reported equivalent or greater medication use and MRU at 1, 2, and 3 months post‐discharge compared with patients with influenza and CRFs. These findings suggest underlying comorbidities have a major impact on the MRU required by adults hospitalized with influenza or RSV.

Despite the substantial disease burden in adults, RSV is primarily recognized as a pediatric pathogen. RSV disease burden and MRU may be underestimated in adults due to lack of routine adult testing, low provider awareness, and the fact that RSV is often not considered for infections resulting in acute exacerbation of underlying respiratory or cardiovascular conditions.^11^ A recent prospective study in adults showed that point‐of‐care RSV testing was associated with reductions in hospital LoS,^38^ possibly due to reduced unnecessary antibiotic use and earlier initiation of supportive therapies.^39^ Improved RSV surveillance among adults is needed to fully evaluate the US RSV burden; the increased use of PCR respiratory pathogen panels in hospitals and emergency departments since the COVID‐19 pandemic began may be useful in this regard.^40^ This could potentially diminish inappropriate antibiotic use, mitigate secondary bacterial infections, and improve outcomes for patients with RSV.

These results support efforts to improve RSV surveillance and diagnostics as well as the development of new agents for RSV prophylaxis and treatment. Development and approval of a vaccine against RSV and improvements in RSV prophylaxis and therapy could yield similar benefits to those derived from influenza therapeutics and vaccines. A recent retrospective modeling study demonstrated that universal influenza vaccination reduces the overall economic burden of influenza in Canada.^41^ Another modeling study estimated that a 5% increase in influenza vaccine efficacy would prevent an additional 1,050,000 influenza infections and 25,000 influenza‐associated hospitalizations annually in the United States.^42^ Moreover, a retrospective US claims‐based cohort study found that influenza antiviral therapy significantly decreased MRU, including hospitalizations, emergency department visits, ICU admissions, and all‐cause total costs.^43^ These studies, in combination with the results reported here, suggest that comparable gains could be made with improved RSV prophylaxis and therapies.

This study provides key information about risk factors and MRU among US adults hospitalized with influenza or RSV and highlights the substantial RSV disease burden in US adults. HARTI was a prospective study conducted over two consecutive epidemic seasons, allowing assessment of disease severity and MRU up to 3 months after hospital discharge. Importantly, the 2017–2019 US epidemic seasons were typical in terms of the number of influenza and RSV cases identified,^44^ and the results of this study were not impacted by the COVID‐19 pandemic; however, it is unclear how the pandemic and its learnings will impact patient susceptibility to influenza and RSV or MRU associated with these viruses. This study has some limitations. Data on supplemental oxygen use prior to hospitalization were only collected for some of the patients enrolled in the study. This makes it difficult to determine the contributions of influenza or RSV disease to oxygen supplementation during hospitalization and post‐discharge. However, baseline supplemental oxygen use was recorded for participants with COPD (influenza: 25.0%; RSV: 52.3%; Table S2). During hospitalization, supplemental oxygen use among all patients with influenza (58.4%) or RSV (76.8%) was much higher than baseline supplemental oxygen use among those with COPD. Another limitation is the small sample size of patients without CRFs, which limits comparisons between those with versus those without CRFs. Finally, clinician‐reported questionnaires were not validated against patients' medical records, and patient‐reported MRU was not validated against medical claims data.

In summary, RSV infection in adults was associated with disease burden and MRU equivalent to or greater than that of influenza. Compared with those with influenza, patients with RSV were older, more likely to have CRFs, and required longer hospital stays; in particular, among patients with CRFs, those with RSV tended to report greater MRU and medication use than those with influenza. These results highlight the substantial, underappreciated disease burden of RSV in the United States and the need for improved RSV surveillance, prophylaxis, and therapies.

## AUTHOR CONTRIBUTIONS

**Jessica Hartnett:** Conceptualization; methodology; validation; writing ‐ reviewing and editing. **Prina Donga:** Conceptualization; methodology; validation; writing ‐ reviewing and editing. **Gabriela Ispas:** Conceptualization; methodology; supervision; validation; writing ‐ reviewing and editing. **Yannick Vandendijck:** Data curation; formal analysis; methodology; software; validation; writing ‐ reviewing and editing. **David Anderson:** Conceptualization; methodology; validation; writing ‐ reviewing and editing. **Stacey House:** Investigation; validation; writing ‐ reviewing and editing. **Selim Suner:** Investigation; validation; writing ‐ reviewing and editing.

### PEER REVIEW

The peer review history for this article is available at https://publons.com/publon/10.1111/irv.12994.

## Supporting information


**Table S1.**Clinical Symptom Scoring Chart^†^
**Table S2.**At‐home Oxygen Supplementation Among Patients with COPD at Baseline
**Figure S1.**Complications during hospitalization

## Data Availability

The data sharing policy of Janssen Pharmaceutical Companies of Johnson & Johnson is available at https://www.janssen.com/clinical-trials/transparency. As noted on this site, requests for access to the study data can be submitted through Yale Open Data Access (YODA) Project site at http://yoda.yale.edu.
