# Supplementary material for: Risk factors and medical resource utilization in US adults hospitalized with influenza or respiratory syncytial virus in the Hospitalized Acute Respiratory Tract Infection study
Source: Influenza Other Respir Viruses. 2022 Apr 26;16(5):906–15. doi: 10.1111/irv.12994 (PMC9343339; doi:10.1111/irv.12994)
Supplement: Supplementary file 1 — Table S1.Clinical Symptom Scoring Chart† Table S2.At‐home Oxygen Supplementation Among Patients with COPD at Baseline Figure S1.Complications during hospitalization [file IRV-16-906-s001.docx]

**Supplementary Materials**

**Supplementary Table 1. Clinical Symptom Scoring Chart^†^**

| **Symptoms** | **Scores** | | | |
| --- | --- | --- | --- | --- |
|  | **0** | **1** | **2** | **3** |
| *General symptoms* | | | | |
| Cough, sputum production, shortness of breath, and malaise | No symptoms | Just noticeable | Bothersome sometimes, not interfering with other activities | Bothersome most of the time, interfering with other activities |
| *Lower respiratory symptoms* | | | | |
| Dyspnea | None | May have brief episodes (minutes to max 1 hour) | May have episodes longer than 1 hour but less than 1 day | May have long episodes (lasting longer than  1 day) |
| Rales, rhonchi, or other abnormal breathing sounds | None | N/A | Scattered wheezes or rhonchi | Widespread wheezes or rhonchi, rales, dyspnea, or signs of consolidation |
| Wheezing | None | Terminal expiration or only with stethoscope | Entire expiration or audible on expiration without stethoscope | Inspiration and expiration without stethoscope |
| *Upper respiratory symptoms* | | | | |
| Nasal discharge | None | Clear, serous, scant but slightly increased | Clear to white, obvious increased volume, minor blood streaks on tissue | Purulent (yellow or green), or gross blood |
| Pharyngitis | None | Mild and/or patchy erythema | Marked and/or confluent erythema | Erythema and purulent exudate |
| Sinus tenderness | None | N/A | Mild tenderness | Severe tenderness or overlying erythema |

N/A, not applicable. **^†^**For patients with any item missing from this survey, the total clinical symptom score is also missing.

**Supplementary Table 2. At-home Oxygen Supplementation Among Patients with COPD at Baseline**

|  | Main study | | Substudy | |
| --- | --- | --- | --- | --- |
|  | **Influenza**  **(n=280)** | **RSV**  **(n=120)** | **Influenza**  **(n=178)** | **RSV**  **(n=112)** |
| COPD presence, n (%) | 84 (30.0) | 44 (36.7) | 57 (32.0) | 40 (35.7) |
| At-home O_2_ use at baseline in the COPD subset, n (%) | 21/84 (25.0) | 23/44 (52.3) | 13/57 (22.8) | 19/40 (47.5) |

COPD, chronic obstructive pulmonary disease; RSV, respiratory syncytial virus.

**Supplementary Figure 1. Complications during hospitalization**
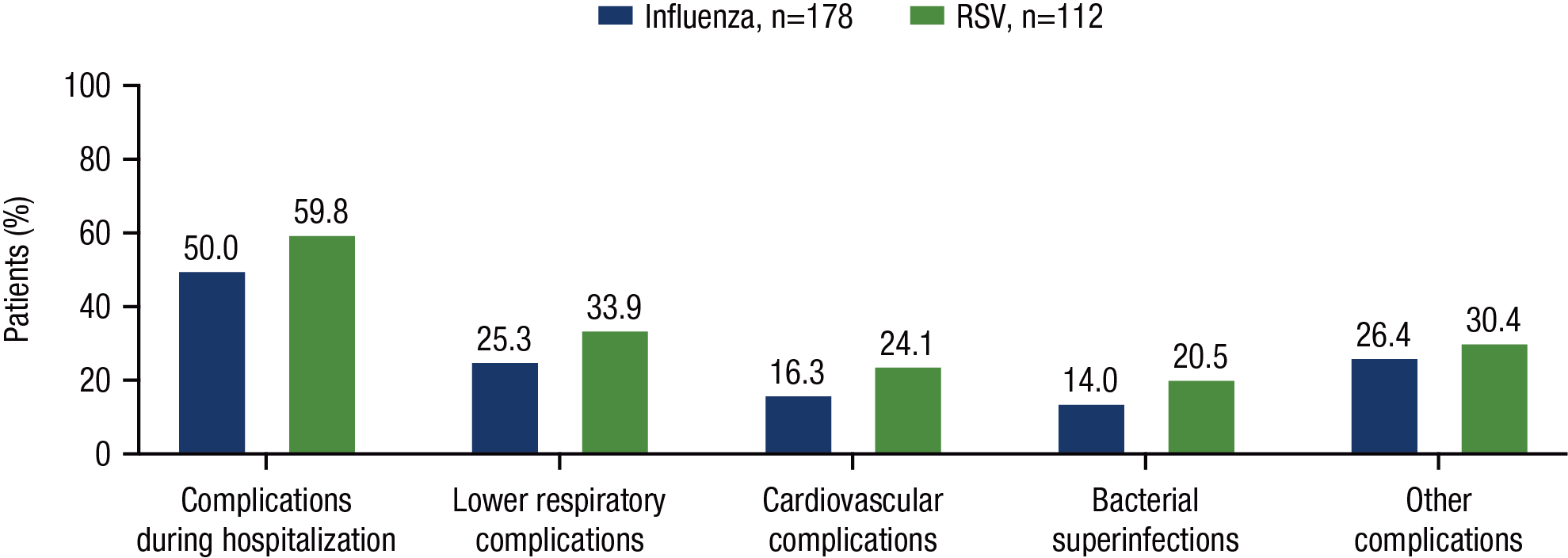


RSV, respiratory syncytial virus.
